# Activation of Complement Components on Circulating Blood Monocytes From COVID-19 Patients

**DOI:** 10.3389/fimmu.2022.815833

**Published:** 2022-02-17

**Authors:** Silvia Lucena Lage, Joseph M. Rocco, Elizabeth Laidlaw, Adam Rupert, Frances Galindo, Anela Kellogg, Princy Kumar, Rita Poon, Glenn W. Wortmann, Andrea Lisco, Maura Manion, Irini Sereti

**Affiliations:** ^1^ HIV Pathogenesis Section, Laboratory of Immunoregulation, National Institute of Allergy and Infectious Diseases, National Institutes of Health, Bethesda, MD, United States; ^2^ AIDS Monitoring Laboratory, Frederick National Laboratory for Cancer Research, Leidos Biomedical Research, Inc., Frederick, MD, United States; ^3^ Clinical Monitoring Research Program Directorate, Frederick National Laboratory for Cancer Research, Leidos Biomedical Research, Inc., Frederick, MD, United States; ^4^ Division of Infectious Diseases and Tropical Medicine, Georgetown University Medical Center, Washington, DC, United States; ^5^ Division of Hospital Medicine at MedStar Georgetown University Hospital, Washington, DC, United States; ^6^ Section of Infectious Diseases, MedStar Washington Hospital Center, Washington, DC, United States

**Keywords:** COVID-19, complement, monocytes, surface expression, inflammation

## Abstract

The coronavirus disease-2019 (COVID-19) caused by the SARS-CoV-2 virus may vary from asymptomatic to severe infection with multi-organ failure and death. Increased levels of circulating complement biomarkers have been implicated in COVID-19-related hyperinflammation and coagulopathy. We characterized systemic complement activation at a cellular level in 49-patients with COVID-19. We found increases of the classical complement sentinel C1q and the downstream C3 component on circulating blood monocytes from COVID-19 patients when compared to healthy controls (HCs). Interestingly, the cell surface-bound complement inhibitor CD55 was also upregulated in COVID-19 patient monocytes in comparison with HC cells. Monocyte membrane-bound C1q, C3 and CD55 levels were associated with plasma inflammatory markers such as CRP and serum amyloid A during acute infection. Membrane-bounds C1q and C3 remained elevated even after a short recovery period. These results highlight systemic monocyte-associated complement activation over a broad range of COVID-19 disease severities, with a compensatory upregulation of CD55. Further evaluation of complement and its interaction with myeloid cells at the membrane level could improve understanding of its role in COVID-19 pathogenesis.

## Introduction

The severe acute respiratory syndrome coronavirus-2 (SARS-CoV-2) is the causative agent of the coronavirus disease-2019 (COVID-19) (1). Clinical outcomes of COVID-19 may vary from asymptomatic or mild disease to severe viral pneumonia with acute respiratory distress syndrome (ARDS) causing high morbidity and mortality (2, 3). Several complications associated with poor prognosis in patients with severe COVID-19 have been described, such as systemic hyperinflammation, respiratory failure, and cardiovascular and thromboembolic complications (3–6). COVID-19-related hyperinflammation and coagulopathy are marked by increased levels of innate inflammatory cytokines, chemokines, and fibrin degradation products (7). Dysregulated activation of the myeloid cell compartment is also increasingly recognized with greater numbers of circulating inflammatory monocytes and increased production of myeloid activation markers (8–10).

The complement system is an important component of innate and adaptive immunity that helps cells opsonize and kill pathogens, directly regulates lymphocyte function, and simultaneously contributes to inflammation and coagulation (11–14). Monocytes and derived macrophages and dendritic cells interact with the complement system through multiple pathways (15). They produce most complement components including the entire C1 complex and express both anaphylatoxin receptors (C3aR, C5aR) and inhibitory receptors (CD55, CD59) (11, 15). Complement has been implicated in the pathogenesis of severe COVID-19 and genetic susceptibility loci that associate with severe disease have been identified in complement genes (16–19). A striking deposition of C5b-9, C4d, and the mannose binding lectin (MBL)-associated serine protease (MASP2) was found in the microvasculature of pulmonary and cutaneous pathologic specimens from COVID-19 patients, suggesting sustained, systemic activation of the complement cascade (20). Co-localization of SARS-CoV-2-specific spike glycoproteins with these complement components in the lung and skin was also observed (20).

Increased levels of complement factors such as C3a, C5a, and C5b-9 have been found in severe COVID-19 when compared to mild infection or healthy controls (21–24). However, differences in systemic complement levels between those with mild-moderate COVID-19 and healthy controls has been less frequently reported (23). Higher levels of C5b-9 and C4d in those presenting with acute COVID-19 have been associated with risk of progression to severe disease and respiratory failure (25). In light of these findings, the use of complement blockade was hypothesized to be potentially efficacious in controlling the hyperinflammation of severe COVID-19 and was trialed in multiple observational studies with mixed results (26–31).

Due to the increasingly recognized role of dysregulated complement activation in COVID-19 related inflammatory complications, further evaluation of its activity at the cell membrane level in different disease severities is important. Here, we assessed both the complement activity and inhibitory receptor expression in circulating blood monocytes in a primarily hospitalized patient cohort with a broad range of disease severity. Our findings provide insights into the role of the complement system in the pathogenesis of COVID-19 showing early activation on monocytes with a potentially compensatory upregulation of the complement inhibitor CD55.

## Materials and Methods

### Study Participants and Approval

Study participants were enrolled in National Institutes of Health (NIH) clinical protocol: COVID-19–associated Lymphopenia Pathogenesis Study in Blood (CALYPSO), NCT04401436 which recruited primarily hospitalized patients at the NIH clinical center, Washington Hospital Center and Georgetown University Hospital between May 2020 and June 2021. Individuals were categorized based on their oxygen requirement at the time of the research blood draw. Cases were originally considered mild if patients required 4 liters of supplemental oxygen or less. Moderate cases required from 4 liters to 50% oxygen concentration (FiO2), and severe cases needed over 50% FiO2 or ICU care. Based on the number of participants included in this study, this classification was changed, and patients were subdivided into two groups: (1) mild-moderate - those requiring 4 liters of supplemental oxygen or less and (2) severe - those who required greater than 4-liters of supplemental oxygen. Only patients who were able to consent themselves were eligible to participate, which excluded enrollment of critically ill ventilated ICU patients. Ten patients were recruited shortly after recovering from COVID-19 (three were follow-ups from acute presentation). Research blood draw for recovered patients was performed at a median of 54-days after symptom onset. De-identified healthy volunteer blood samples were obtained on NIH IRB-approved protocol 99-CC-0168. All participants provided written informed consent prior to any study procedures in accordance with the Declaration of Helsinki.

### Cell Culture and Treatments

Cryopreserved ficoll-isolated peripheral blood mononuclear cells (PBMCs) from patients or healthy control individuals (HCs) were thawed and resuspended in RPMI-1640 media (Corning, NY, USA) supplemented with 10% heat-inactivated human AB serum (Gemini Bio-Products, West Sacramento, USA) and 0.05% benzonase (MilliporeSigma, USA). Cells were rested for 1 hour at 37°C and 5% CO_2_ and subsequently plated at 10^6^ cells/well in round bottom 96-well plates (Corning Costar™, MilliporeSigma, USA) followed by immune staining.

### Flow Cytometry

PBMCs were incubated with LIVE/DEAD Fixable AQUA Dead Cells Stain (Thermo Fisher, USA) for 15 min at RT, followed by extracellular staining in Gelatin Veronal Buffer (GVB) (CompTech complement Technology Inc, Tyler, TX) for 30 min at room temperature (RT) to characterize complement staining in monocytes with the following monoclonal antibodies (clones) and fluorochromes: CD14 (M5E2) BV605, CD16 (3G8) PE-Cy7, CD56 (HCD56) BV421, CD66b (G10F5) Pacific Blue from Biolegend, HLA-DR (L243) APC-Cy7, C3 (1H8) PE and CD59 (p282(H19)) BV711 from BD bioscience, CD2 (RPA-2.10) eFluor 450, CD3 (UCHT1) eFluor 450, CD19 (SJ25C1) eFluor 450 and CD20 (2H7) eFluor 450 from Life Technology and, finally, C1q (polyclonal) FITC from Dako. Data were acquired on a BD Fortessa flow cytometer (BD Biosciences). All compensation and gating analyses were performed using FlowJo 10.5.3 (TreeStar, Ashland, OR, USA).

### Serum and Plasma Biomarker Measurements

Plasma and serum samples from patients were used to measure the following biomarkers according to the manufacturer’s instructions. Plasma levels of inteleukin-6 (IL-6), IL-8, IL-10, IL-27, tumor necrosis factor-α (TNFα), C-reactive protein (CRP), and serum amyloid A (SAA) were quantified using ELISA kits from Meso Scale Discovery platforms (Gaithersburg, MD, USA). Serum levels of C3a, C5a, circulating immune complexes-C1q (CIC-C1q), CIC-C3d, C1q, D-dimer, and ferritin were also measured with ELISA Meso Scale Discovery platforms (Gaithersburg, MD, USA). Plasma levels of sIL-6Ra were quantified by ELISA from R&D Systems (Minneapolis, MN, USA).

### Statistical Analyses

Statistical analyses were performed using non-parametric Mann-Whitney test in GraphPad Prism 8.0.1 software (GraphPad, USA). Data are presented as median with interquartile ranges. Spearman correlation matrix was performed to create heatmap. Differences were considered significant when *p* < 0.05.

## Results

### Patient Characteristics

The study enrolled 14 healthy controls and 49 COVID-19 patients, primarily hospitalized across three academic institutions. The cohort included only three outpatients and therefore was subdivided based on degree of hypoxia. If patients had normal oxygen saturation or required minimal supplemental oxygen (<4-liters) they were considered mild-moderate disease (n=29). For comparison, individuals in this mild-moderate cohort met ACTT-1 ordinal scale criteria (32) for group-2 (n=3), group-3 (n=13), and group-4 (n=13). Individuals requiring higher levels of supplemental oxygen were classified as severe disease (n=13) in our cohort and met ACTT-1 ordinal scale criteria for group-5 (n=11) or group-6 (n=2). Only two of these patients required mechanical ventilation and one died. Seven participants were recruited after recovery from COVID-19 and eight patients that were enrolled during acute infection had follow-up samples drawn as part of the recovered group (n=15). The average age of the cohort was 51.4-years and 23 (47%) were women. Twenty-four participants (49%) were African American and 20 (41%) were Caucasian. Additional patient characteristics are reported in **Table 1**.

**Table 1:** Patient characteristics by disease severity.

Characteristic	Mild-Moderate	Severe	Recovered
**N**	30	12	15
**Age – y**	55.5 (42.3-62.7)	56.5 (47.3-62.5)	52 (37-58.5)
**Female – no. (%)**	14 (47)	6 (50)	10 (66.7)
**BMI (Kg/m2)**	32.6 (29-39)	36.5 (28.5-41.3)	31 (27.2-44.5)
**Race – no. (%)**			
**White**	13 (43)	2 (17)	8 (53.3)
**African American**	15 (50)	8 (66)	5 (33.3)
**Other**	2 (7)	2 (17)	2 (13.3)
**Total neutrophil count**	3.1 (2.0-4.7)	7.3 (5.5-7.5)	3.0 (2.5-4.4)
**Total lymphocyte count**	1.3 (1.0-1.7)	1.1 (0.6-1.6)	1.6 (1.4-2.2)
**Time from symptom onset (Days) at sampling**	8.5 (5-11.8)	12 (8-15.8)	79 (51-142)
**Comorbidities – no. (%)**			
**Hypertension**	15 (50)	8 (67)	5 (33.3)
**Diabetes mellitus**	12 (40)	7 (58)	2 (13.3)
**COPD/Asthma**	5 (17)	4 (33)	2 (13.3)
**Renal Disease**	3 (10)	2 (17)	0 (0)
**Treatment – no. (%)**			
**Remdesivir**	3 (10)	8 (67)	2 (13.3)
**Dexamethasone**	4 (13)	8 (67)	3 (20)

### Monocytes From COVID-19 Patients Display Higher Membrane Expression of Distinct Complement Components

**Figure 1:**
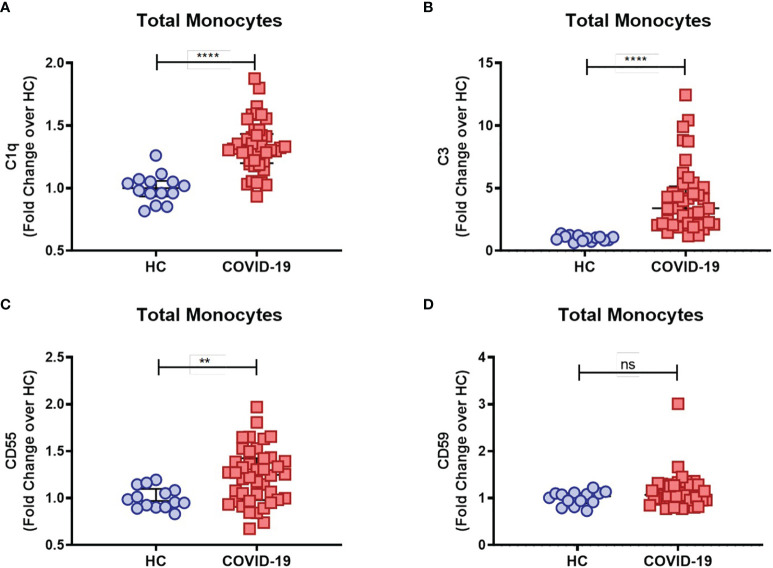
Complement deposition on circulating blood monocytes during acute COVID-19. The mean fluorescence intensity (MFI) of C1q **(A)**, C3 **(B)**, CD55 **(C)** and CD59 **(D)** expression on circulating blood monocytes was compared between healthy controls (HC) (n = 14) and COVID-19 patients (n = 42) by flow cytometry and presented as Fold change over their respective experimental HC mean, and graphs depict median and interquartile range. Data were analyzed using the Mann-Whitney test. ****P < 0.0001; **P < 0.01; *ns*, not significant.

COVID-19 severity has been associated with dysregulated activation and recruitment of circulating blood monocytes leading to pathological inflammation and tissue damage (9). Although primarily targeting pathogen cell surfaces, deposition of the classical complement sentinel C1q and C3 may overcome protective complement regulators in bystander host cells, therefore resulting in activation of the complement cascade on their surface (33). We further characterized complement activation in COVID-19 patients by measuring the membrane-bound complement components on circulating monocytes. We found that COVID-19 patients had increased levels of monocyte membrane-bound C1q (mC1q) (**Figure 1A**) and C3 (mC3) (**Figure 1B**) when compared to healthy control individuals (HCs).

As briefly mentioned above, in order to prevent damaging activation on host cell surfaces while preserving regulatory functions of activated complement components, several complement regulators are distributed as cell surface-bound inhibitors, such as CD55, which accelerates the decay of C3 and C5 convertases, and CD59, which binds to the C5b-8 complex, thus preventing full MAC assembly [reviewed in Refs (33, 34)]. Interestingly, we found elevated CD55 (**Figure 1C**) but not CD59 (**Figure 1D**) monocyte surface expression levels in COVID-19 patients when compared to the control group, consistent with a compensatory mechanism in the setting of persistent complement activation.

**Figure 2:**
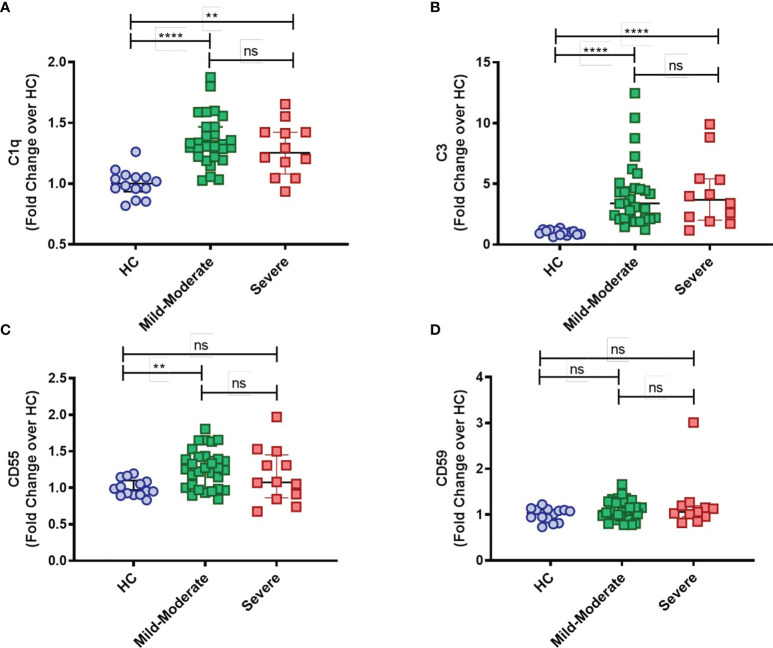
Complement deposition on circulating blood monocytes from COVID-19 stratified by disease status. The mean fluorescence intensity (MFI) of C1q **(A)**, C3 **(B)**, CD55 **(C)** and CD59 **(D)** expression on circulating blood monocytes was compared among healthy controls (HC) (n = 14), COVID-19 Mild to Moderate patients (n = 30) and Severe patients (n = 12) by flow cytometry. Data are presented as Fold change over their respective experimental HC mean, and graphs depict median and interquartile range. Data were analyzed using the Kruskal-Wallis test. **P < 0.01, ****P < 0.0001; *ns*, not significant.

Furthermore, while mC1q upregulation was more prominent within the classical CD14^high^CD16^-^ and intermediate CD14^high^CD16^+^ monocytes than in the patrolling CD14^low^CD16^+^ group (**Supplementary Figure 1B**), mC3 is shown to be broadly distributed among the distinct monocyte subsets (**Supplementary Figure 1A**). Interestingly, expression of both CD55 and CD59 membrane-bound complement inhibitors was found specifically elevated in intermediate CD14^high^CD16^+^ monocytes from COVID-19 patients when compared to HCs (**Supplementary Figures 1C, D**, respectively). None of these markers, however, was associated with disease severity, since similar surface expression of mC1q, mC3, CD55 and CD59 was observed when COVID-19 patients were grouped in mild-moderate *versus* severe disease (**Figures 2A**–**D**).

**Figure 3:**
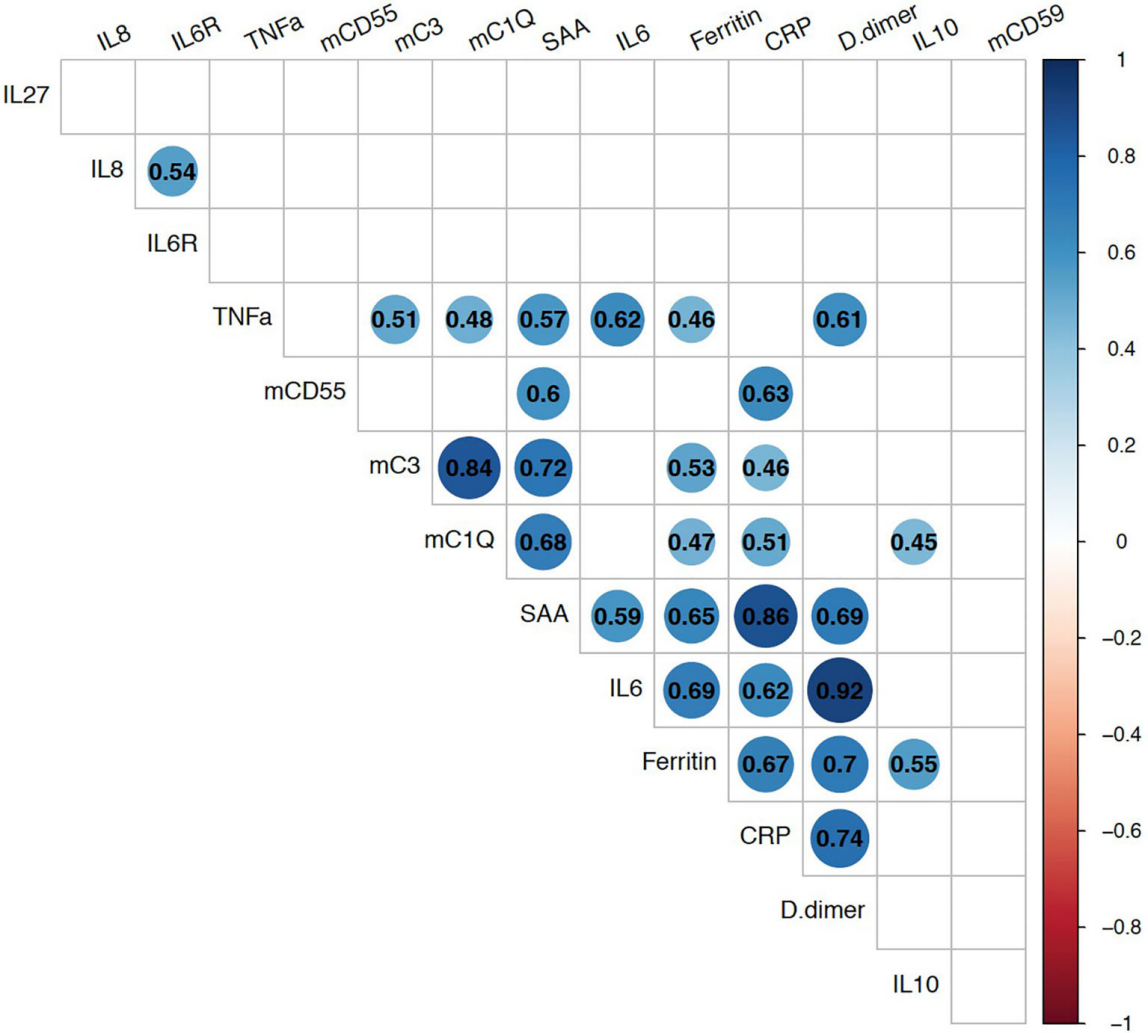
Relationship between monocyte bound complement markers and circulating systemic inflammatory biomarkers during acute COVID-19. Multi-parameter Spearman’s correlation analysis of monocyte membrane-bound markers (mC1q, mC3, mCD55, mCD59) and plasma circulating biomarkers from COVID-19 patients (statistically significant associations with p < 0.05 are highlighted with correlation coefficients in colored circles).

We next evaluated the relationship of these membrane-bound complement markers with innate immune biomarkers which had been checked in 22-participants as part of a previously completed study (35). Spearman correlation analyses showed strong positive association between mC1q and mC3 (**Figure 3**). Both mC1q and mC3 also had significant positive correlation with CRP, SAA, TNFα, and ferritin. CD55 also correlated with CRP and SAA, while CD59 did not have a significant relationship with any immune biomarkers.

Finally, we sought to investigate whether altered complement surface expression on monocytes from COVID-19 patients persists after recovery (median 54-days, IQR: 46-78 days) by comparing those markers across recovered individuals and HCs. We observed that a small but statistically significant increase of C1q and C3 complement deposition persisted in recovered COVID-19 patients over 6-weeks after infection suggesting residual complement activity. There were similar expression levels of complement inhibitory receptors between COVID-19 recovered patients and HCs consistent with the initial increase in CD55 returning to normal levels post-recovery from COVID-19 (**Supplementary Figures 2A**–**D**).

## Discussion

Severe COVID-19 is characterized by increases in innate immune signaling, myeloid activation, and endothelial dysfunction (8, 36–38). We demonstrate in a cohort of primarily hospitalized patients with acute COVID-19 that there is increased complement deposition on the cell surface of monocytes with a possibly compensatory upregulation of the CD55 complement inhibitor. Increased circulating complement biomarkers have been consistently reported in severe disease and positively correlate with CRP and D-dimer (22, 23). High expression of complement receptors (C3aR1 and C5aR1) has also been found on blood and pulmonary myeloid cells (21, 39). Increased complement activation in less severe COVID-19 has been less frequently described (23). Soluble complement biomarkers are rapidly degraded which could limit their sensitivity in detecting subtle changes during mild-moderate infections. In addition, complement is tightly regulated, and detectable increases may only occur after the breakdown of key regulatory pathways in severe disease.

In our cohort, which primarily included mild-moderate cases, we evaluated complement activation at the cellular level using flow cytometry and identified increases in mC3 and mC1q on monocytes. Measuring cellular levels of complement components may reflect immunoregulatory function associated with early complement activation rather than full activation of the complement cascade. Alternatively, the level of mC3 and mC1q can reflect membrane translocation of monocyte expressed complement components upon their activation.

Activated circulating blood monocytes have been reported as major sources of complement C1q (40). Self-made C1 complex (C1q, C1s, C1r) can translocate to the monocyte cell surface and activate the complement cascade (41), thus suggesting a possible role for monocyte-driven classical complement activation during COVID-19. In addition, our findings of increased levels of mC3 on monocytes could indicate external complement deposition and overactivation of the alternative pathway (AP) in COVID-19. In agreement with that, Yu et al. have demonstrated the SARS-CoV-2 spike (S) protein binds heparan sulfate *in vitro*, thus activating the AP on cell surfaces, with deposition of C3 fragments and C5b-9 on target cells (42). We cannot rule out, however, that other complement pathways could trigger the cascade, as C3 deposition provides an amplification loop once complement is initiated by any of the three pathways. In this regard, it has been shown that deposition of the mannose binding lectin (MBL)-associated serine protease MASP2 occurs in the microvasculature of the lung and skin of COVID-19 patients, suggesting systemic activation of the Lectin pathway (LP) (20). In fact, the SARS-CoV-2 S and N-proteins were also found to bind MBL with subsequent LP-mediated C3b and C4b deposition.

C1q and C3 expression was most prominent on classical (CD14^high^CD16^-^) and intermediate (CD14^high^CD16^+^) inflammatory monocytes which are known to be expanded during acute SARS-CoV-2 infection (9, 10, 35). No apparent relation with clinical severity was revealed, likely because our cohort primarily recruited hospitalized patients with variable oxygen requirements although few had severe disease requiring mechanical ventilation limiting the sensitivity of this comparison.

Membrane bound C1q and C3 were strongly associated with each other consistent with these markers reflecting systemic complement activity. They were also positively associated with other inflammatory biomarkers markers including CRP, SAA, TNFα, and ferritin. As complement is closely involved in innate immune activation, these results further support the involvement of the complement system in the control of early phases of SARS-CoV-2 infection.

Undesired complement activity on bystander host cells has been shown to occur in acute and chronic disease states due to a dysregulated inflammatory response (33, 43). It has been shown that inflammatory monocyte-derived macrophages are prevalent in the lungs during SARS-CoV-2 infection, and they play a major role in the production of inflammatory mediators (44, 45). Our findings build on these prior studies and highlight the contribution of inflammatory monocytes to complement activation during COVID-19-related inflammation. These results have implications for the utilization of complement inhibition in the management of COVID-19 which has been trialed in observational studies with variable results as reviewed by Deravi et al. (46). Our findings support activation of the classical and alternative complement pathways on monocytes even in mild-moderate SARS-CoV-2 infection with a compensatory upregulation of CD55. Detailed characterization of the breakdown of immunoregulatory pathways leading to severe disease is critical to identify predictive markers that could indicate who would benefit most from therapeutic complement inhibition and if C3 or C5 specific inhibitors could be of greater utility. It is likely the complement system is important in combating acute COVID-19 and therefore, identifying the optimal timing of treatment is key to avoid diminishing the acute immune response against replicating SARS-CoV-2.

Changes in the expression of complement inhibitory receptors remains unexplored in COVID-19. We identified significant increases in CD55 but not CD59 on monocytes in acute SARS-CoV-2 infection. This increase was most prominent on intermediate (CD14^high^CD16^+^) monocytes. No difference was found between those with mild-moderate vs severe infection. Positive associations were noted between CD55 and CRP and SAA, whereas no significant associations with CD59 and innate inflammatory markers was found suggesting that this inhibitory receptor may be playing a less important role in monocyte regulation of the complement system in acute COVID-19. However, upregulation of the CD55 inhibitory receptor may play a vital role in controlling complement activation and preventing the dramatic increases seen in those with severe COVID-19. Interestingly, polymorphisms causing functional variation in CD55 have been implicated in severe influenza infection (47). Identifying individuals with a genetic predisposition to severe disease could also help risk stratify those most likely to benefit from therapeutic complement inhibition. Prior studies have identified variants in complement-associated genes that incur greater risk of severe disease (17–19). Further study of the CD55 regulatory pathway is required to characterize its involvement in limiting systemic complement activation and hyperinflammation during respiratory virus infections.

Interestingly, we identified that increased mC1q and mC3 deposition on monocytes persisted for over 6-weeks after SARS-CoV-2 infection when compared to healthy controls. COVID-19 patients even with mild disease can continue to experience prolonged symptoms after recovery from acute infection, and mechanisms contributing to this post-acute COVID-19 syndrome are unknown (48, 49). In a previous study, we described persistent inflammasome activation and caspase-1 activity in monocytes could be detected even after a short recovery from acute COVID-19 (35). Our current results build on these findings identifying residual increased complement activation persisting on monocytes after a short recovery period. Our cohort was too small to identify associations between residual complement activity and lingering clinical symptoms, but this potential mechanism should be explored in future studies.

Limitations of this study include the relatively small sample size and the variability from symptom onset to research blood collection between the groups. Our cohort primarily included hospitalized patients with COVID-19 which allowed us to compare complement activation across a spectrum of oxygen requirements. Individuals in this cohort primarily met ACTT-1 ordinal scale criteria between group-3 (inpatient but no hypoxia) to group-5 (requiring high flow oxygen supplementation). This did limit our sensitivity to detect differences between mild outpatient infections and severe disease requiring mechanical ventilation. The biomarker data were originally collected for a prior COVID-19 study and results were not available for each participant (35).

In conclusion, we provide further evidence of increased complement activity across SARS-CoV-2 infection severities. Greater cellular expression and/or deposition of complement markers on monocytes occurs in acute COVID-19 and is associated with a concomitant rise in CD55 but not CD59 expression. This increase in inhibitory receptor expression could serve as a vital regulatory mechanism preventing the pathologic levels of complement activation described in severe SARS-CoV-2 infections. Further evaluation of regulatory complement pathways is essential to understand their role in limiting the hyperinflammatory sequelae of COVID-19 and could aid in the development of new immunomodulatory therapies to prevent these complications.

## Data Availability Statement

The original contributions presented in the study are included in the article/**Supplementary Material**. Further inquiries can be directed to the corresponding authors.

## Ethics Statement

Study participants were enrolled in the NIH clinical protocol: COVID-19-associated Lymphopenia Pathogenesis Study in Blood (CALYPSO), NCT04401436, which recruited at the NIH clinical center, Washington Hospital Center and Georgetown University Hospital. Healthy volunteer blood samples were obtained under the protocol 99-CC-0168 and were de-identified prior to distribution. Protocols in this study were reviewed and approved by the National Institutes of Health (NIH) Central Intramural Institutional Review Board (IRB). All participants provided written informed consent prior to any study procedures in accordance with the Declaration of Helsinki. The patients/participants provided their written informed consent to participate in this study.

## Author Contributions

SL, JR, and IS were involved in conception and design of the study. SL, JR, and AR performed experiments and/or data analysis. JR, EL, FG, AK, PK, RP, GW, AL, MM, and IS recruited patients, assisted in clinical care, and helped with data curation. AR and IS provided resources and reagents. SL, JR, and IS were involved in drafting the manuscript. All authors have reviewed and approved the final manuscript.

## Funding

This project was supported in part by the intramural research program of NIAID/NIH, with federal funds from the National Cancer Institute, National Institutes of Health, under the following Contract Numbers. (HHSN261200800001E, HHSN2612015000031 or 75N910D00024). The content of this publication does not necessarily reflect the views or policies of the Department of Health and Human Services, nor does mention of trade names, commercial products, or organizations imply endorsement by the U.S. Government.

## Conflict of Interest

Authors AR and AK were employed by Leidos Biomedical Research, Inc.

The remaining authors declare that the research was conducted in the absence of any commercial or financial relationships that could be construed as a potential conflict of interest.

## Publisher’s Note

All claims expressed in this article are solely those of the authors and do not necessarily represent those of their affiliated organizations, or those of the publisher, the editors and the reviewers. Any product that may be evaluated in this article, or claim that may be made by its manufacturer, is not guaranteed or endorsed by the publisher.
