# Supplementary figures and images for: Activation of Complement Components on Circulating Blood Monocytes From COVID-19 Patients
Source: Front Immunol. 2022 Feb 17;13:815833. doi: 10.3389/fimmu.2022.815833 (PMC8892247; doi:10.3389/fimmu.2022.815833)

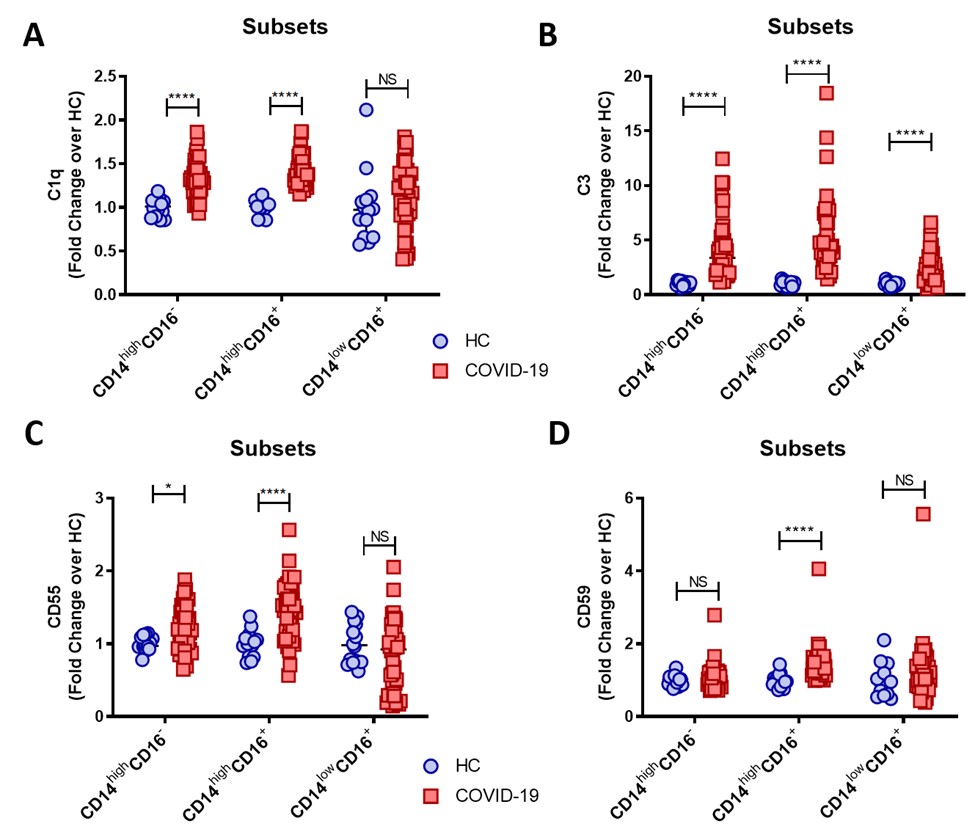

Supplement: Supplementary file 1 [file Image_1.jpeg]

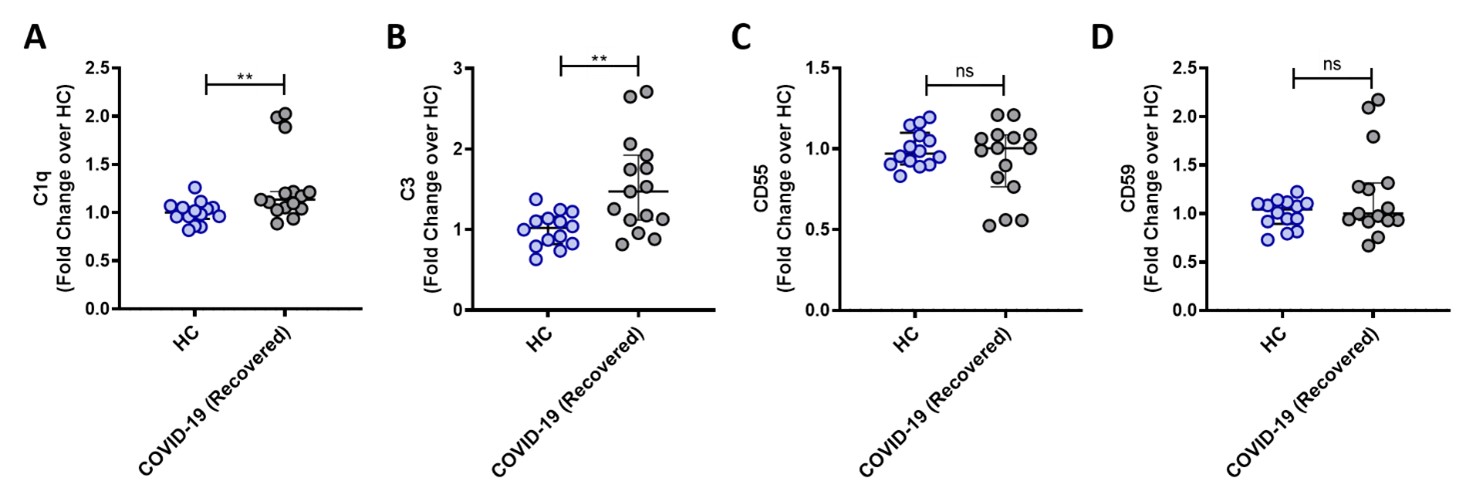

Supplement: Supplementary file 2 [file Image_2.jpeg]
